# Antibiotic Resistance and Biofilm Gene Distribution in Colistin‐Resistant *Acinetobacter baumannii*

**DOI:** 10.1002/mbo3.70332

**Published:** 2026-06-18

**Authors:** Zainab Amer Hatem, Fadhela Nafaa Kafe, Farkad Hawas Musa, Sarah F. Al‐Taie, Nabaa Hisham Ateya, Leqaa Majeed Aziz, Erta Rajabi, Raad N. Hasan

**Affiliations:** ^1^ Department of Biotechnology, College of Science University of Diyala Baqubah Iraq; ^2^ Medical Laboratory Techniques Department, College of Health and Medical Technology University of Al‐Maarif Anbar Iraq; ^3^ Department of Biology, College of Education for Pure Sciences University of Anbar Ramadi Iraq; ^4^ Department of Biotechnology, College of Science University of Baghdad Baghdad Iraq; ^5^ Biotechnology Department, College of Applied Science Fallujah University Fallujah Iraq; ^6^ College of Medicine University of Fallujah Fallujah Iraq; ^7^ Faculty of Medicine Tehran University of Medical Sciences Tehran Iran; ^8^ Biotechnology and Environmental Center University of Fallujah Fallujah Iraq

**Keywords:** *Acinetobacter baumannii*, antibiotic resistance, biofilm, colistin, oxacillinase

## Abstract

*Acinetobacter baumannii*, a multidrug‐resistant opportunistic bacterium, poses a substantial hazard in hospital settings. The emergence of colistin‐ and tigecycline‐resistant strains further limits treatment options and necessitates detailed investigation of resistance mechanisms. A total of 144 clinical *A. baumannii* isolates from multiple hospitals in Iran were identified using standard microbiological and molecular techniques. Antimicrobial susceptibility was assessed using both disk diffusion and broth microdilution techniques. Biofilm formation was quantified by crystal violet staining. Resistance and biofilm‐related genes were detected by conventional polymerase chain reaction (PCR). The expression of key resistance genes (*pmrA*, *pmrB*, *adeB*, *adeJ*, and *adeG*) was evaluated by quantitative PCR (qPCR) in resistant isolates, and MLST was performed to determine the genetic relatedness among tigecycline‐ and colistin‐resistant isolates. Resistance to colistin and tigecycline was observed in 3 (2.08%) and 2 (1.4%) isolates, respectively, and 90.9% of the isolates were biofilm producers, with higher odds of strong biofilm formation significantly correlating with the presence of *bla*_PER1_. All isolates carried *pmrA* and *pmrB*, but only colistin‐resistant isolates showed overexpression of these genes compared to susceptible ones. MLST revealed diverse sequence types among resistant isolates, including ST188, ST138, ST387, ST2288, and ST3337. This study highlights the complex interplay between the presence of genes, their expression, and the resistance phenotype in *A. baumannii* and underscores the importance of monitoring chromosomal resistance determinants for effective control and treatment strategies.

## Graphical abstract

Multicenter analysis of 144 clinical *Acinetobacter baumannii* isolates in Iran revealed high MDR/XDR rates and widespread biofilm formation (90.9%). Colistin resistance was linked to pmrAB overexpression, while blaPER‐1 was independently associated with strong biofilm production, highlighting chromosomal resistance mechanisms and biofilm as key drivers of persistence and treatment failure.

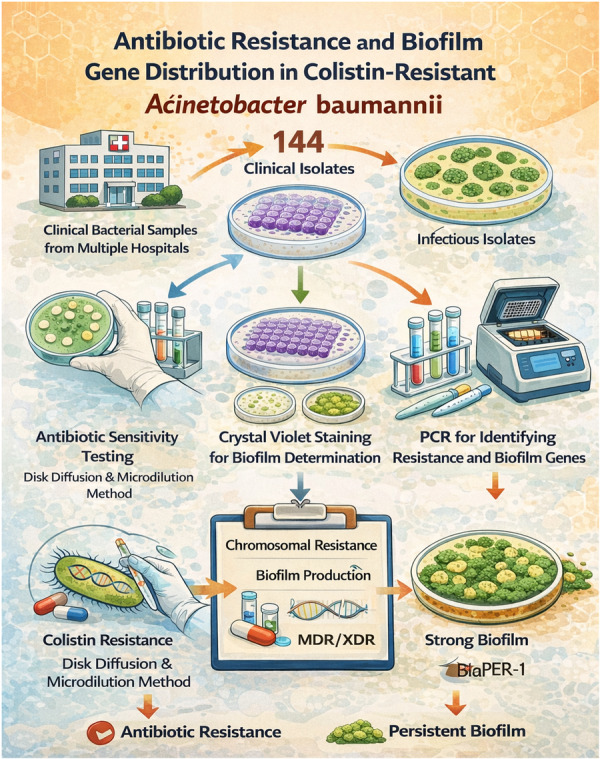

**Abbreviations**
*A. baumannii*
: *Acinetobacter baumannii*
*bap*
: biofilm‐assisted protein
CLSI: Clinical and Laboratory Standards Institute
*csuE*
: chaperon‐usher‐pilus
EUCAST: European Committee on Antimicrobial Susceptibility Testing
FDA: Food and Drug Administration
LB: lysogeny broth
LPS: lipopolysaccharide
*mcr*
: mobilized colistin resistance
MDR: multi‐drug resistant
MIC: minimum inhibitory concentration
MLST: multi‐locus sequence typing
OD: optical density
*ompA*
: outer membrane protein A
O‐F test: oxidation–fermentation test
PCR: polymerase chain reaction
XDR: extensively drug resistant

## 1 Introduction

Initially thought to be a low‐grade pathogen, *Acinetobacter baumannii* (*A. baumannii*) has recently been identified as an opportunistic nosocomial pathogen with a wide range of clinical symptoms, to which few antibiotics remain effective, as several studies have demonstrated *A. baumannii* strains are resistant to carbapenems, aminoglycosides, and in some cases, colistin (Mea et al. 2021; Ibrahim et al. 2021; da Silva et al. 2018; Tafreshi et al. 2019).

Carbapenem resistance in *A. baumannii* strains can be primarily due to intrinsic or acquired *bla*_*OXA*_ genes encoding Ambler class D *β*‐lactamases (oxacillinases), among which *OXA‐23‐like*, *OXA‐24/40‐like*, *OXA‐51‐like*, and *OXA‐58‐like* groups are more predominant, while rarer variants, including *OXA‐143‐like* and *OXA‐235‐like*, have also been reported worldwide (Yazdansetad et al. 2019; Thirapanmethee et al. 2020; Yousefi Nojookambari et al. 2021; Abbasi et al. 2021). Colistin resistance arises through processes such as lipopolysaccharide (LPS) complete loss or lipid A modification by phosphoethanolamine via *pmrC*, which is activated by mutations in *pmrA* and *pmrB*, in addition to resistance induction via plasmid‐containing mobilized colistin resistance (*mcr*) genes (Srisakul et al. 2022; Novović and Jovčić 2023). Additionally, *Acinetobacter* spp. resistance to tigecycline is associated with increased *AdeABC*, *AdeIJK*, *AdeFGH*, *AbeM*, and *AdeDE* pump activity and the presence of the *tet(X)* gene, as mutations in the nucleotides or amino acids of the *AdeRS* system can lead to overexpression of *adeABC* and confer resistance to tigecycline (Ni et al. 2014; Montaña et al. 2015). Furthermore, the *BaeSR* system promotes tigecycline resistance by positively regulating *adeA* and *adeB* expression (Lin et al. 2014). In addition to antibiotic resistance mechanisms, several studies have demonstrated a positive correlation between biofilm development and antibiotic resistance in *A. baumannii* isolates, which is mediated by pili production and assembly and is encoded by the chaperon‐usher pilus *(Csu)/BABCDE* chaperon‐usher system, facilitating surface adhesion (Ibrahim et al. 2021; Tomaras et al. 2003). Furthermore, the *csuE* gene was found to be highly prevalent within multi‐drug resistant (MDR) *A. baumannii* clinical isolates, with certain reports indicating a prevalence as high as 100% (Zeighami et al. 2019; Mendes et al. 2023; Yang et al. 2019). Other genes involved in biofilm formation include biofilm‐associated protein (*bap*) and the outer membrane protein A (*ompA*), all essential to epithelial adhesion and biofilm production (Roy et al. 2022; Brossard and Campagnari 2012; Gaddy et al. 2009; Kwon et al. 2017).

The investigation of *A. baumannii* resistance and its association with biofilm formation may yield more effective treatment strategies, enhanced infection control measures, and the identification of novel therapies to combat this pathogen, underscoring the necessity of understanding the molecular mechanisms that govern resistance to last‐resort antibiotics such as tigecycline and colistin (Gedefie et al. 2021). Consequently, the present study sought to explore the presence and expression of critical antibiotic resistance genes alongside biofilm‐associated genes, as well as the genotype–phenotype association between resistance‐associated genes and biofilm formation to enhance the understanding of the pathogenic capacity of *A. baumannii* in clinical settings in Iran.

## 2 Materials and Methods

### 2.1 Bacterial Isolates

**Figure 1:**
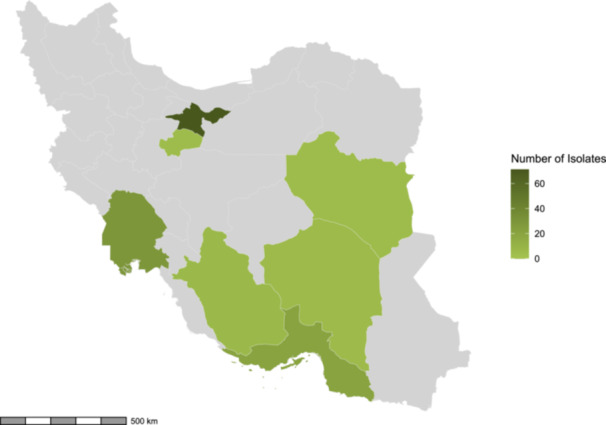
Distribution of isolates across Iran's provinces.

*A. baumannii* isolates were obtained from different hospitalized patients in Iran from Tehran (*n* = 71), Ahvaz (*n* = 29), Bandar Abbas (*n* = 21), Kerman (*n* = 6), Shiraz (*n* = 7), Birjand (*n* = 5), and Qom (*n* = 5) over 16 months from February 2023 to August 2024 (Figure 1). Samples were retrieved from blood, sputum, tracheal aspirate, wound, and abscess, which were then transported to the laboratory. Isolates were identified in the laboratory using standard biochemical tests for bacterial identification, including oxidase and catalase tests, growth at 44°C, motility and urease test, and oxidation–fermentation tests (O‐F tests) (CLSI 2025). Consequently, isolates were validated as *A. baumannii* by using the *blaOXA‐51‐like* sequencing with specific primers (Table S1) (Ahmad and Mohammad 2020). The *A. baumanii* ATCC 19606 was used as quality control for this test.

### 2.2 Antibiotic Susceptibility Testing

The antibiotic susceptibility of the isolates was evaluated using the Kirby–Bauer disk diffusion method using 10 disks, including ceftazidime (30 µg), piperacillin/tazobactam (10 µg), tetracycline (30 µg), amikacin (30 µg), ciprofloxacin (5 µg), imipenem (10 µg), meropenem (10 µg), and gentamicin (120 μg) (Mast, Company). The results were evaluated according to the criteria of the Clinical and Laboratory Standards Institute (CLSI) (CLSI 2025). Minimum inhibitory concentrations (MICs) for colistin and tigecycline susceptibility were assessed via the broth microdilution test. The CLSI removed the classification of *Acinetobacter* spp. as colistin‐susceptible, retaining only intermediate (MIC ≤ 2 µg/L) and resistant (MIC ≥ 4 µg/L) categories, but the European Committee on Antimicrobial Susceptibility Testing (EUCAST) still categorizes isolates with a colistin MIC ≤ 2 µg/L as colistin‐susceptible (Giske et al. 2022). The Food and Drug Administration (FDA) used breakpoints for tigecycline susceptibility, categorizing it into susceptible (MIC ≤ 2 µg/L), intermediate (4 µg/L), and resistant (MIC ≥ 8 µg/L) levels (Curcio and Fernández 2007). Reference strains *Escherichia coli* ATCC 25922 and *Pseudomonas aeruginosa* ATCC 27853 were used as quality controls.

### 2.3 Biofilm Formation Assay

Biofilm formation was measured using a modified microtiter plate assay (Kobayashi et al. 2021). The isolates were cultivated in Tripticase Soy Broth (TSB, Merck, Germany) with 0.5% glucose (Merck, Germany) and incubated at 37°C under static conditions and without CO_2_ supplementation for the whole night. After diluting the cultures 1:40 in TSB with 0.5% glucose, 200 μL of the diluted solution was applied to each well of a polystyrene plate. The plates were incubated at 37°C for 48 h. The negative control wells contained 200 μL of TSB with 0.5% glucose. Following three slow washes with PBS (pH 7.2; Invitrogen, USA), the wells were fixed for 20 min with methyl alcohol (Merck, Germany), dried at room temperature (20°C–25°C), and then filtered using 0.1% safranin (Merck, Germany). In each well, 150 μL of 95% ethyl alcohol (Merck, Germany) was used to dissolve the safranin dye that was attached to the adhesive cells. The ELISA reader (BioTek, USA) was then used to measure the optical density (OD) of each well at 490 nm (A490). The definition of optical density cut‐off (ODc) was 3 times SD above the mean OD of the negative control. The absorbance of the safranin‐stained adherent cells was used to examine and categorize the biofilm development with strains. Biofilm formation was defined using the following means of OD values: non‐biofilm producer (OD ≤ 0.059), weak biofilm producer (0.059 < OD ≤ 0.118), medium biofilm producer (0.118 < OD ≤ 0.236), and strong biofilm producer (OD > 0.236) (Ghasemi et al. 2018).

### 2.4 DNA Extraction

Isolates were incubated on lysogeny broth (LB) for 24 h at 37°C, and genomic DNA was extracted using the High Pure polymerase chain reaction (PCR) Template kit (GeNet Bio Company, Daejeon, Korea; Cat. No, K‐3000) according to the manufacturer's guidelines. The total DNA concentration was determined using a Nanodrop instrument (Thermo Scientific, USA).

### 2.5 Polymerase Chain Reaction (PCR) for Antibiotic Resistance and Biofilm Genes

Biofilm‐related genes (*bap*, *csuE*, and *ompA*), colistin resistance‐related genes (*pmrA*, *pmrB, mcr‐1*, and *mcr‐2*), tigecycline resistance‐related genes (IS*Aba1*, *tet(X)*, and *tet(39)*), class A *β*‐lactamase gene (*bla*_*PER‐1*_), and class D oxacillinase genes (*bla*_*OXA‐51*_, *bla*_*OXA‐23*_, *bla*_*OXA‐24*_, and *bla*_*OXA‐58*_) were detected using PCR using the primers demonstrated in Table S1. Each reaction mixture contained 1 µM of each primer, 3 µL of extracted DNA, 12.5 µL of 2× red PCR master mix (Amplicon, Denmark), and 7.5 µL of H_2_O. The protocol was applied by peqStar (Peqlab, Germany): initial denaturation at 94°C for 5 min, followed by 36 cycles of denaturation at 94°C for 45 s, annealing at 55°C–62.5°C for 30 s, extension at 72°C for 45 s, and final extension at 72°C for 5 min. PCR products were electrophoresed on a 1%–1.5% agarose gel, visualized using DNA‐safe staining, and photographed under ultraviolet light.

### 2.6 Expression of *pmrA*, *pmrB*, *adeB*, *adeJ*, and *adeG*

Colistin‐ and tigecycline‐resistant and susceptible isolates were evaluated for expression of *pmrA*, *pmrB*, *adeB*, *adeJ*, and *adeG* genes. Cell suspensions were prepared and cultured on LB broth. After overnight growth, total RNA was extracted from the cell suspensions using the RNX‐Plus Kit (Cat. No. RN7713C, Sinaclon, Iran) according to the manufacturer's instructions. The contaminating DNA was eliminated by RNase‐free DNase I (Fermentas, USA). RNA concentration and quality assessment were based on measuring OD (at 260 nm wavelength) using a spectrophotometer (Denovix DS‐11, USA). DNase‐treated RNA was reverse‐transcribed into cDNA using the Takara Kit (Japan). The primers used for real‐time PCR are shown in Table S1. Real‐time PCR assay was performed on synthesized cDNA using the Power SYBR Green PCR Master Mix (Bioneer, Korea) on a Corbett Rotor‐Gene 6000 real‐time rotary analyzer (Corbett Life Science, Australia). Each amplification protocol included initial denaturation at 95°C for 5 min, 40 cycles of denaturation at 95°C for 30 s, annealing at 55°C for 30 s, and extension at 72°C for 30 s. The expression level of each gene was normalized using the *rpoB* housekeeping gene and was calculated based on the 2^−ΔΔCT^ method. *A. baumannii* ATCC 19606 was used as the reference strain (Khoshnood et al. 2020).

### 2.7 Multi‐Locus Sequence Typing (MLST)

MLST was performed on three colistin‐resistant isolates and two tigecycline‐resistant isolates. Internal regions of seven housekeeping genes—*gltA* (citrate synthase), *gyrB* (DNA gyrase subunit B), *gdhB* (glucose dehydrogenase B), *recA* (homologous recombination factor), *cpn60* (60‐kDa chaperonin), *gpi* (glucose‐6‐phosphate isomerase), and *rpoD* (RNA polymerase σ70 factor)—were amplified using PCR. Primer sequences used from the PubMLST website are listed in Table S1. The PCR program consisted of 30 cycles: 1 min of denaturation at 94°C, 1 min of annealing at 55°C, and 2 min of extension at 72°C, preceded by a denaturation at 94°C for 2 min and followed by an extension at 72°C for 2 min (Bartual et al. 2005). In total, 9.5 μL of distilled water, 1 μL of DNA, 1 μL of each forward and reverse primer, and 12.5 μL of 2× PCR Master Mix (Ampliqon, Denmark) were used in the PCR reactions. The PCR products were purified using a PCR purification kit (Bioneer Co., Korea), and then nucleotide sequencing of the amplicons was performed using an ABI PRISM 3700 sequencer (Macrogen Co., Korea). The sequenced data obtained was viewed in Chromas version 1.45 software. Additionally, sequence alignment was performed using the Nucleotide BLAST program. The *A. baumannii* MLST database was used to give distinct allele numbers to the sequences, and the allelic sequences from the seven genes were combined to create the allelic profile for each isolate (Jolley et al. 2018).

### 2.8 Statistical Analysis

Descriptive data were analyzed using R (version 4.4.1), and categorical and continuous variables were reported as frequencies and mean (SD), respectively. The Cochran–Armitage test (*DescTools* package) was used to evaluate the trends in biofilm formation, antibiotic resistance patterns, and virulence gene distribution across the ordered biofilm levels (no, weak, moderate, and strong biofilm formation). Univariate proportional odds models (*ordinal* package) and multivariate proportional odds models (*VGAM* package), adjusted for sample source and region, assessed gene–biofilm associations. A *p* value < 0.05 indicated statistical significance. Visualization was performed using the *ggplot2* and *sf* packages, with city‐level polygon data extracted from the geoBoundaries project (https://data.humdata.org/dataset/geoboundaries-admin-boundaries-for-iran-islamic-republic-of).

## 3 Results

### 3.1 Bacterial Isolates

A total of 144 nonduplicate isolates of *A. baumannii* were obtained from various hospitals in Iran, comprising 71 (49.3%) male and 73 (50.7%) female patients with a mean ± SD age of 56.35 ± 14 years. These isolates were predominantly recovered from sputum (*n* = 89, 61.8%), followed by blood (*n* = 29, 20.1%), tracheal aspirate (*n* = 18, 12.5%), wound (*n* = 7, 4.9%), and abscess (*n* = 1, 0.7%). The demographics and characteristics of the participants are summarized in Table 1.

**Table 1:** Baseline characteristics of the participants.

Patients (*n* = 144)	*N* (%)
Gender	
Female	73 (50.7%)
Male	71 (49.3%)
Mean age ± SD^a^	56.35 ± 14
City of sample collection	
Birjand	5 (3.5%)
Ahvaz	29 (20.1%)
Shiraz	7 (5%)
Qom	5 (3.4%)
Kerman	6 (4.1%)
Sample sources	
Blood	29 (20.1%)
Sputum	89 (61.8%)
Tracheal aspirate	18 (12.5%)
Wound	7 (4.9%)
Abscess	1 (0.7%)
Resistance profile	
MDR^b^	96 (66.7%)
XDR^c^	47 (32.6%)

^a^ SD = standard deviation,

^b^ MDR = multi‐drug resistant.

^c^ XDR = extensively drug‐resistant.

### 3.2 Antibiotic Susceptibility Testing

According to the CLSI interpretation criteria, all of the isolates exhibited resistance to ceftazidime and piperacillin/tazobactam (*n* = 144, 100%), followed by meropenem (*n* = 118, 81.9%), imipenem (*n* = 113, 78.5%), amikacin (*n* = 96, 66.7%), gentamicin (*n* = 84, 58.3%), tetracycline (*n* = 76, 52.8%), and ciprofloxacin (*n* = 65, 45.1%). Conversely, according to EUCAST and FDA MIC breakpoints, resistance to colistin (*n* = 3, 2.08%) and tigecycline (*n* = 2, 1.4%) was detected at notably low levels. The MIC ranges, MIC_50_, MIC_90_, and the frequency of resistant, intermediate, and susceptible isolates are shown in Table 2. Furthermore, 66.7% (*n* = 96) and 32.6% (*n* = 47) of our isolates were MDR and extensively drug resistant (XDR), respectively (Table 1).

**Table 2:** *A. baumannii* clinical isolates' antimicrobial susceptibility.

Antimicrobial agents	MIC^a^ (µg/mL)			Disc diffusion number (%)		
	Range	MIC_50_	MIC_90_	Susceptible	Intermediate	Resistant
Ceftazidime	—	—	—	0 (0)	0 (0)	144 (100)
Imipenem	—	—	—	31 (21.5)	0 (0)	113 (78.5)
Meropenem	—	—	—	26 (18.1)	0 (0)	118 (81.9)
Ciprofloxacin	—	—	—	79 (54.9)	0 (0)	65 (45.1)
Piperacillin/tazobactam	—	—	—	0 (0)	0 (0)	144 (100)
Gentamicin	—	—	—	54 (37.5)	6 (4.2)	84 (58.3)
Amikacin	—	‐‐‐	—	40 (27.8)	8 (5.6)	96 (66.7)
Colistin	≤ 2–≥ 4	0.5	0.25	141 (97.9)	0 (0)	3 (2.08)
Tetracycline	—	—	—	56 (38.9)	12 (8.3)	76 (52.8)
Tigecycline	≤ 4–≥ 16	1	0.5	142 (98.6)	0 (0)	2 (1.4)

^a^ MIC = minimum inhibitory concentration.

### 3.3 Biofilm‐Forming Ability

**Figure 2:**
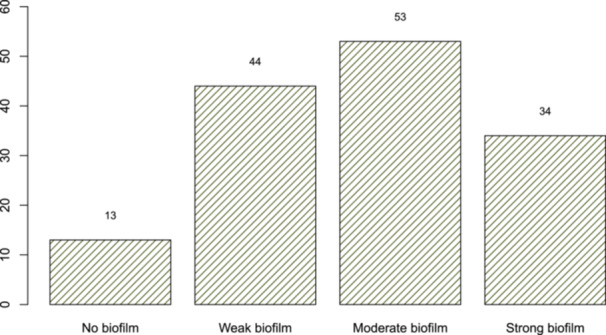
Biofilm formation distribution levels among the *A. baumannii* isolates.

The biofilm assay revealed that among the 144 tested isolates, 44 (30.5%) were weak biofilm producers, 53 (36.8%) were of moderate biofilm‐producing capacity, 34 (23.6%) isolates were strong biofilm producers, and 13 (9%) were non‐biofilm producers (Figure 2).

### 3.4 Presence of Antibiotic‐Resistant Genes

The presence of the *bla*_*OXA‐51*_, *pmrA*, and *pmrB* genes was detected in all 144 isolates. The distribution of other genes was as follows: 38 (26.4%), 122 (84.7%), 34 (23.6%), and 31 (21.5%) of isolates were positive for the *bla*_*PER‐1*_, *bla*_*OXA‐23*_, *bla*_*OXA‐24*_, and *bla*_*OXA‐58*_, respectively, and *mcr‐1* and *mcr‐2* genes were detected in neither of the isolates (Table 3). *tet(X)*, IS*Aba1*, and *tet(39)* were detected in 18 (12.5%), 128 (88.9%), and 17 (11.8%) of isolates, respectively, and all tigecycline‐resistant isolates carried all three of these tigecycline‐resistance genes (Table 4).

**Table 3:** Prevalence of resistance‐ and biofilm‐associated genes among *A. baumannii* isolates.

Gene	Frequency (%)
*bap*	114 (79.2)
*csuE*	127 (88.2)
*ompA*	107 (74.3)
*mcr‐1*	0 (0)
*mcr‐2*	0 (0)
*pmrA*	144 (100)
*pmrB*	144 (100)
*bla*_*PER‐1*_	38 (26.4)
*bla*_*OXA‑51*_	144 (100)
*bla*_*OXA‑23*_	122 (84.7)
*bla*_*OXA‑24*_	34 (23.6)
*bla*_*OXA‑58*_	31 (21.5)
*tet(X)*	18 (12.5)
*tet(39)*	17 (11.8)
IS*Aba1*	128 (88.9)

**Table 4:** Association between the biofilm formation strength and the distribution of genes.

Genes		Biofilm formation strength				
		No biofilm	Weak	Moderate	Strong	*p* c
*bap*	−a	4 (13.3%)	14 (46.7%)	10 (33.3%)	2 (6.7%)	**0.005**
	+^b^	9 (7.9%)	30 (26.3%)	43 (37.7%)	32 (28.1%)	
*csuE*	−	1 (5.9%)	8 (47.1%)	2 (11.8%)	6 (35.3%)	0.943
	+	12 (9.4%)	36 (28.3%)	51 (40.2%)	28 (22%)	
*ompA*	−	1 (2.7%)	13 (35.1%)	13 (35.1%)	10 (27%)	0.376
	+	12 (11.2%)	31 (29%)	40 (37.4%)	24 (22.4%)	
*bla*_*PER‐1*_	−	12 (11.3%)	41 (38.7%)	40 (37.7%)	13 (12.3%)	**< 0.001**
	+	1 (2.6%)	3 (7.9%)	13 (34.2%)	21 (55.3%)	
*bla*_*OXA‑51*_	−	0 (0%)	0 (0%)	0 (0%)	0 (0%)	—
	+	13 (9%)	44 (30.6%)	53 (36.8%)	34 (23.6%)	
*bla*_*OXA‑23*_	−	0 (0%)	6 (27.3%)	8 (36.4%)	8 (36.4%)	0.058
	+	13 (10.7%)	38 (31.1%)	45 (36.9%)	26 (21.3%)	
*bla*_*OXA‑24*_	−	13 (11.8%)	30 (27.3%)	39 (35.5%)	28 (25.5%)	0.914
	+	0 (0%)	14 (41.2%)	14 (41.2%)	6 (17.6%)	
*bla*_*OXA‑58*_	−	9 (8%)	34 (30.1%)	42 (37.2%)	28 (24.8%)	0.347
	+	4 (12.9%)	10 (32.3%)	11 (35.5%)	6 (19.4%)	
*mcr‐1*	−	13 (9%)	44 (30.6%)	53 (36.8%)	34 (23.6%)	—
	+	0 (0%)	0 (0%)	0 (0%)	0 (0%)	
*mcr‐2*	−	13 (9%)	44 (30.6%)	53 (36.8%)	34 (23.6%)	—
	+	0 (0%)	0 (0%)	0 (0%)	0 (0%)	
*pmrA*	−	0 (0%)	0 (0%)	0 (0%)	0 (0%)	—
	+	13 (9%)	44 (30.6%)	53 (36.8%)	34 (23.6%)	
*pmrB*	−	0 (0%)	0 (0%)	0 (0%)	0 (0%)	—
	+	13 (9%)	44 (30.6%)	53 (36.8%)	34 (23.6%)	
*tet(X)*	−	10 (7.9%)	35 (27.8%)	50 (39.7%)	31 (24.6%)	**0.039**
	+	3 (16.7%)	9 (50%)	3 (16.7%)	3 (16.7%)	
IS*Aba1*	−	0 (0%)	6 (37.5%)	8 (50%)	2 (12.5%)	1
	+	13 (10.2%)	38 (29.7%)	45 (35.2%)	32 (25%)	
*tet(39)*	−	10 (7.9%)	40 (31.5%)	46 (36.2%)	31 (24.4%)	0.438
	+	3 (17.6%)	4 (23.5%)	7 (41.2%)	3 (17.6%)	

*Note:* Bold values indicate statistical significance at *p* < 0.05.

^a^ − = negative.

^b^ + = positive.

^c^ Cochran–Armitage test was employed.

### 3.5 Association Between Biofilm Formation Ability and Genes' Presence

**Figure 3:**
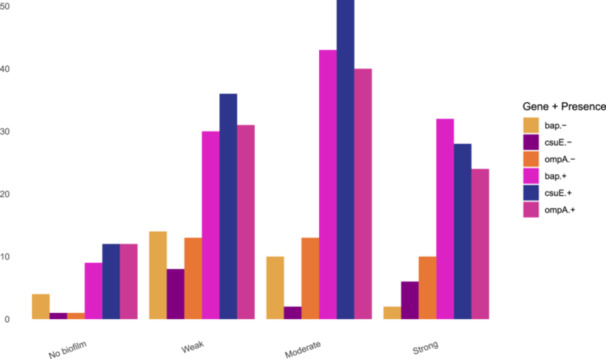
Distribution of biofilm‑related genes across biofilm formation strengths.

Among the isolates, the detection rates of *bap*, *ompA*, and *csuE* were 114 (79.1%), 107 (74.3%), and 127 (88.2%), respectively. Particularly, isolates positive for biofilm‐related genes most commonly produced moderate‐strength biofilms (Table 4 and Figure 3). Initial analyses on the association between genes' presence and biofilm formation strength revealed significant associations between biofilm formation ability and the *bap* (*p* = 0.005), *tet(X)* (*p* = 0.039), and *bla*_*PER‐1*_ genes (*p* < 0.001). Further exploration using univariate proportional odds regression suggested that isolates with the *bla*_*PER‐1*_ gene had significantly higher odds of stronger biofilm formation (OR = 8.63; 95% CI = 4.02–19.44; *p* < 0.001), as did those with the *bap* gene (OR = 2.88; 95% CI = 1.39–6.07; *p* = 0.005), while a negative association was found with *tet(X)* (OR = 0.36; 95% CI = 0.14–0.90; *p* = 0.030). However, following adjustments for region and sample source, only *bla*_*PER‐1*_ remained significant (AOR = 9.64; 95% CI = 4–23.25; *p* < 0.001) (Table 5).

**Table 5:** Univariate and multivariate ordinal logistic regression of biofilm formation strength on biofilm‑associated genes.

Genes	Univariate analysis^d^				Multivariate analysis^e^			
	OR^b^	95% CI^a^		*p*	AOR^c^	95% CI		*p*
		Upper bound	Lower bound			Upper bound	Lower bound	
*csuE*	1.03	0.39	2.71	0.953	—	—	—	—
*ompA*	0.78	0.39	1.53	0.466	—	—	—	—
*bap*	2.88	1.39	6.07	**0.005**	2.09	0.21	4.63	0.07
*bla*_*PER‐1*_	8.63	4.02	19.44	**< 0.001**	9.64	4	23.25	**< 0.001**
*bla*_*OXA‑23*_	0.47	0.20	1.07	0.073	—	—	—	—
*bla*_*OXA‑24*_	0.96	0.49	1.88	0.897	—	—	—	—
*bla*_*OXA‑58*_	0.72	0.34	1.48	0.367	—	—	—	—
*tet(X)*	0.36	0.14	0.90	**0.030**	0.57	0.21	1.55	0.271
IS*Aba1*	1.06	0.43	2.57	0.900	—	—	—	—
*tet(39)*	0.73	0.29	1.86	0.514	—	—	—	—

*Note:* Bold values indicate statistical significance at *p* < 0.05.

^a^ CI = confidence interval.

^b^ OR = odds ratio.

^c^ AOR = adjusted odds ratio.

^d^ Univariate proportional odds ordinal logistic regression.

^e^ Multivariate proportional odds ordinal logistic regression.

### 3.6 Distribution of Antibiotic Resistance Across Biofilm Formation Levels

**Figure 4:**
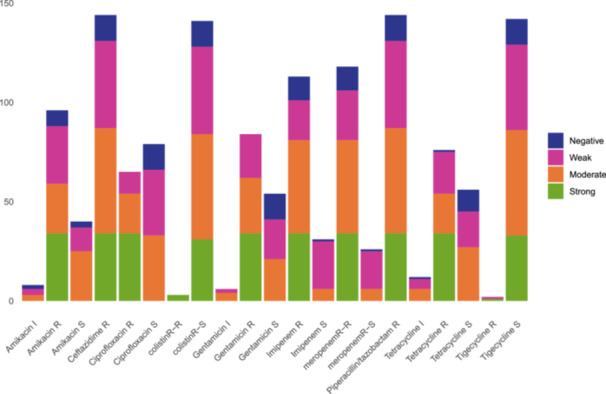
Distribution of biofilm formation strengths across antibiotic resistance profiles.

The majority of ceftazidime‐ and piperacillin/tazobactam‐resistant isolates were moderate biofilm producers. While tetracycline‐, amikacin‐, ciprofloxacin‐, imipenem‐, meropenem‐, and gentamicin‐resistant isolates predominantly produced strong biofilms. All of three colistin‐resistant isolates formed strong biofilms, and tigecycline‐resistant isolates exhibited varying levels of biofilm formation: one isolate was classified as a weak biofilm producer and one as a strong biofilm producer. Detailed demonstrations of biofilm production capability distribution across isolates are provided in Table S3 and Figure 4.

### 3.7 Expression of *pmrAB* and *adeB*, *adeJ*, and *adeG* Genes

Real‐time PCR analysis was used to measure the expression of *pmrA* and *pmrB* in colistin‐resistant and tigecycline‐resistant isolates, as well as those resistant to *adeB, adeG, and adeJ*, compared to susceptible ones. As demonstrated in Table S2, the *AdeB* gene was overexpressed (53.8–49.23‐fold) in two isolates, while overexpressions of *adeJ* (7.29‐fold) and *adeG* (8.84‐fold) were detected in one isolate. The *pmrA* genes were overexpressed in all colistin‐resistant isolates. However, the overexpression of the *pmrB* gene (4.85‐fold) was detected in one isolate.

### 3.8 MLST Analysis

According to the results obtained from the analysis of MLST data, the sequence types (STs) of colistin‐resistant isolates include ST188, ST138, and ST387. In contrast, the ST types of tigecycline‐resistant isolates include ST2288 and ST3337. The STs and genetic characteristics patterns of colistin‐ and tigecycline‐resistant *A. baumannii* isolates are illustrated in Table S2.

## 4 Discussion

In the last decade, attentions have shifted to *A. baumannii* as a particularly important pathogen due to its ability to acquire resistance to a wide range of antibiotics (Ayoub Moubareck and Hammoudi Halat 2020). The findings of the present study demonstrated substantially high resistant rates to almost all tested antibiotics, except for colistin and tigecycline. Although Ghamari et al.'s (2024) study revealed a similar pattern, suggesting high rates of antibiotic resistance against ceftazidime, meropenem, imipenem, piperacillin/tazobactam, and ciprofloxacin, the number of colistin‐resistant isolates reported in their study was higher than that in the present study, with this discrepancy possibly attributed to variances in study design, as the present study involved a multicenter approach with 3.3 times more isolates. Contrary to Ghamari et al.'s study, a prospective multicenter study reported comparable resistant rates of both colistin (1.2%) and tigecycline (1.7%), although colistin‐resistant isolates were higher in the present study (2.08%) (Boral et al. 2019). Overall, our findings support the administration of a colistin‐or‐tigecycline‐based regimen, particularly in cases of MDR infection (Chuang et al. 2014).

Despite the relatively high MDR and XDR isolates identified in the current study, our XDR isolates were considered more susceptible compared to a recent Iranian study among patients with coronavirus disease 2019 (91%), while alarmingly high rates of MDR isolates were identified (Ghamari et al. 2024). Similarly, Mirzaei et al. (2020) reported MDR trends close to our findings, while their XDR pathogens were higher than that reported in the present study. These discrepancies could reflect sampling variations, as both studies were region‐centered compared to this multicenter study. Furthermore, it is worth noting that since early 2016, a national antimicrobial stewardship program (ASP) in Iran was introduced as a solution to reducing antimicrobial resistance, possibly justifying the lower rates of XDR isolates detected in this study compared to previous studies, as it was conducted across various hospitals in which ASP protocols may be more implemented (Salehi et al. 2025).

Tigecycline resistance is commonly driven by *adeABC* (efflux pump) overexpression, *tet(x)* family inactivating enzymes, and *tet(39)* (Chen et al. 2024; Lucaßen et al. 2021; Hamidian et al. 2016); accordingly, overexpression of *adeB*, *tet(x)*, and *tet(39)* was observed in our tigecycline‐resistant isolates. Furthermore, neither the *mcr‐1* nor the *mcr‐2* genes, which are determinants of plasmid‐mediated colistin resistance, were found in the colistin‐resistant isolates; this aligns with global data indicating their sparse detection in *A*. *baumannii* isolates, as Rahman et al. observed a 20% rate of the *mcr‐1* gene in their MDR isolates, while Hameed et al. revealed a rate of 1.6% (Rahman and Ahmad 2019; Hameed et al. 2019). Additionally, *pmrAB*, which is related to polymyxin resistance by regulating genes associated with lipid A modification, was detected in all isolates (Ko et al. 2023). The response regulator, *pmrA*, is phosphorylated by *pmrB*, a sensor histidine kinase, which then induces conformational changes in *pmrA*, resulting in stimulation of target genes' transcription, which are implicated in resistance (Ouyang et al. 2024). Additionally, *pmrA*'s and *pmrB*'s overexpression was identified in three colistin‐resistant isolates in the present study, per the findings of Khoshnood et al. reporting their elevated expression levels in colistin‐resistant isolates, and further corroborated by the *pmrAB* two‐component system in Park et al.'s study (Khoshnood et al. 2020; Park et al. 2011). However, an Iranian study reported that *pmrA* gene expression was higher in colistin‐resistant strains, while *pmrB* gene expression remained unchanged (Sepahvand et al. 2017). Comparably, *tet(x)* and *tet* (Lucaßen et al. 2021 *)* both confer resistance to tetracycline antibiotics, both of which were similarly distributed across our isolates, and *bla*_*OXA‐51*_ and *bla*_*OXA‐23*_ were recently reported to be the most common oxacillinases in *A. baumannii* globally, with varying prevalence rates across different geographic locations, which is consistent with our findings (Özkul and Hazırolan 2021; Li et al. 2019; Wang et al. 2018).

In addition to genetic‐mediated antibiotic resistance, the capacity to form biofilm is one of the most significant factors related to the pathogenesis of *A. baumannii* (Monfared et al. 2019; Vijayakumar et al. 2016). As expected, our findings demonstrated that 90.9% of isolates were biofilm producers of varying strengths, with the majority classified as moderate‐ and weak‐biofilm producers, which aligns with previous studies where 100%, 72.9%, and 89.47% of *A. baumannii* isolates were identified as biofilm producers in two Iranian studies and one Nepalese study (Monfared et al. 2019; Khoshnood et al. 2023; Ghimire et al. 2021). Furthermore, our findings demonstrated a significant association between the presence of the *bla*_*PER‐1*_ gene and higher odds of stronger biofilm formation, as reported by Monafred et al. who similarly found a significant correlation between *bla*_*PER‐1*_ and the ability to form biofilms, suggesting that carriers of *bla*_*PER‐1*_ may also have an enhanced capacity for biofilm formation (Monfared et al. 2019). Additionally, although three previously published studies revealed significant differences in biofilm formation abilities among *A. baumannii* isolates carrying the *bap* gene compared to those lacking *bap*, further analyses in the present study indicated such an association was confounded by covariates such as region and sample source (Park et al. 2011; Bardbari et al. 2017; Sung et al. 2016).

Using MLST, our findings revealed that the STs of colistin‐resistant isolates include ST188, ST138, and ST387, and the STs of tigecycline‐resistant isolates include ST2288 and ST3337. In a study conducted in China, 74 clinical isolates of *A. baumannii* resistant to tigecycline were examined via MLST, identifying 10 different sequence types and 8 novel types, with ST208 and ST191 identified as the dominant STs (Deng et al. 2014). Palmieri et al. also reported that utilizing the MLST technique with the Pasteur template, it was determined that the majority of the strains belonged to two main global clones, GC1 and GC2. However, by using the University of Oxford's MLST template, they were able to analyze the GC2 strains more closely and categorize them into five types (ST425, ST208, ST195, ST451, and ST436) (Palmieri et al. 2019). In a study by Mustapha et al. 42 isolates from various individuals were analyzed, including 21 pairs of colistin‐resistant and ‐susceptible strains, and phylogenetic and MLST analysis revealed that some colistin‐resistant isolates were genetically highly similar to susceptible isolates from the same patient, indicating that resistance evolved in vivo. In contrast, several other resistant isolates exhibited significant genetic variations from susceptible isolates, which were most likely caused by reinfection with different strains (Mustapha et al. 2018).

Lastly, we would like to acknowledge the limitations of the present study; first, the small number of isolates used for MLST analysis restricted our ability to broadly conclude the genetic diversity of the isolates. Furthermore, financial and logistical constraints precluded the application of whole genome sequencing (WGS), limiting deeper exploration of molecular resistance mechanisms, and lastly, the predominance of sputum‐derived isolates may have introduced sampling bias. Thus, future studies should incorporate larger, multicenter collections and employ WGS‐based approaches to better elucidate the molecular resistance pathways inducing antimicrobial resistance in *A. baumannii* isolates.

## 5 Conclusion

In conclusion, the present multicenter study provides updated insights into the antimicrobial resistance profiles, biofilm‐forming capacity, and genetic characteristics of *A. baumannii* isolates recovered from hospitalized patients in Iran, as the high prevalence of MDR and XDR phenotypes, combined with the presence of resistance genes such as *bla*_*PER‐1*_, *bap*, and *tet(x)*, underscore the critical importance of antimicrobial stewardship, infection control measures, and continuous molecular surveillance to mitigate the ongoing threat posed by MDR/XDR isolates. Furthermore, biofilm formation was highly prevalent, and the distribution of biofilm‑associated genes revealed that *bla*_*PER‑1*_ remained independently associated with stronger biofilm formation even after adjustment for region and sample source, suggesting that this gene may serve as a marker of successful biofilm‑forming lineages circulating in hospital settings.

## Author Contributions

**Zainab Amer Hatem:** conceptualization, data curation, investigation, methodology, resources, software, writing – original draft, writing – review and editing. **Fadhela Nafaa Kafe:** conceptualization, data curation, investigation, methodology, resources, software, writing – original draft, writing – review and editing. **Farkad Hawas Musa:** conceptualization, data curation, investigation, methodology, resources, software, writing – original draft, writing – review and editing. **Sarah F. Al‐Taie:** methodology, resources, software, writing – original draft, writing – review and editing. **Nabaa Hisham Ateya:** methodology, resources, software, writing – original draft, writing – review and editing. **Leqaa Majeed Aziz:** project administration, data curation, writing – review and editing. **Erta Rajabi:** project administration, data curation, writing – review and editing. **Raad N. Hasan:** data curation, methodology, project administration, resources, software, supervision, visualization, writing – review and editing.

## Funding

The authors have nothing to report.

## Ethics Statement

The study was conducted in accordance with the ethical principles of the 1964 Helsinki Declaration and its later amendments or comparable ethical standards. The study protocol and ethical considerations were approved by the Ethics Committee of the Islamic Azad University, North Tehran Branch, Tehran, Iran.

## Consent

Participants were informed of the objectives of this study and signed a written informed consent form before their participation.

## Conflicts of Interest

The authors declare no conflicts of interest.

## Supporting information

Supporting File 1

## Data Availability

The data sets used and/or analyzed during the current study are available from the corresponding author upon reasonable request.

## References

[mbo370332-bib-0001] Abbasi, E. , H. Goudarzi , A. Hashemi , et al. 2021. “Decreased carO Gene Expression and OXA‐Type Carbapenemases Among Extensively Drug‐Resistant *Acinetobacter baumannii* Strains Isolated From Burn Patients in Tehran, Iran.” Acta Microbiologica et Immunologica Hungarica 68, no. 1: 48–54. 10.1556/030.2020.01138 32365048

[mbo370332-bib-0002] Ahmad, N. H. , and G. A. Mohammad . 2020. “Identification of *Acinetobacter baumannii* and Determination of MDR and XDR Strains.” Baghdad Science Journal 17, no. 3: 0726.

[mbo370332-bib-0003] Ayoub Moubareck, C. , and D. Hammoudi Halat . 2020. “Insights Into *Acinetobacter baumannii*: A Review of Microbiological, Virulence, and Resistance Traits in a Threatening Nosocomial Pathogen.” Antibiotics 9, no. 3: 119. 32178356 10.3390/antibiotics9030119 PMC7148516

[mbo370332-bib-0004] Bardbari, A. M. , M. R. Arabestani , M. Karami , F. Keramat , M. Y. Alikhani , and K. P. Bagheri . 2017. “Correlation Between Ability of Biofilm Formation With Their Responsible Genes and MDR Patterns in Clinical and Environmental *Acinetobacter baumannii* Isolates.” Microbial Pathogenesis 108: 122–128. 28457900 10.1016/j.micpath.2017.04.039

[mbo370332-bib-0005] Bartual, S. G. , H. Seifert , C. Hippler , M. A. Luzon , H. Wisplinghoff , and F. Rodríguez‐Valera . 2005. “Development of a Multilocus Sequence Typing Scheme for Characterization of Clinical Isolates of *Acinetobacter baumannii* .” Journal of Clinical Microbiology 43, no. 9: 4382–4390. 16145081 10.1128/JCM.43.9.4382-4390.2005 PMC1234098

[mbo370332-bib-0006] Boral, B. , Ö. Unaldi , A. Ergin , R. Durmaz , and Ö. K. Eser . 2019. “A Prospective Multicenter Study on the Evaluation of Antimicrobial Resistance and Molecular Epidemiology of Multidrug‐Resistant *Acinetobacter baumannii* Infections in Intensive Care Units With Clinical and Environmental Features.” Annals of Clinical Microbiology and Antimicrobials 18, no. 1: 19. 31266519 10.1186/s12941-019-0319-8 PMC6607529

[mbo370332-bib-0007] Brossard, K. A. , and A. A. Campagnari . 2012. “The *Acinetobacter baumannii* Biofilm‐Associated Protein Plays a Role in Adherence to Human Epithelial Cells.” Infection and Immunity 80, no. 1: 228–233. 22083703 10.1128/IAI.05913-11 PMC3255684

[mbo370332-bib-0008] Chen, X. , Y. Li , Y. Lin , et al. 2024. “Comparison of Antimicrobial Activities and Resistance Mechanisms of Eravacycline and Tigecycline Against Clinical *Acinetobacter baumannii* Isolates in China.” Frontiers in Microbiology 15: 1417237. 39380684 10.3389/fmicb.2024.1417237 PMC11458409

[mbo370332-bib-0009] Chuang, Y. C. , C. Y. Cheng , W. H. Sheng , et al. 2014. “Effectiveness of Tigecycline‐Based Versus Colistin‐ Based Therapy for Treatment of Pneumonia Caused by Multidrug‐Resistant *Acinetobacter baumannii* in a Critical Setting: A Matched Cohort Analysis.” BMC Infectious Diseases 14: 102. 24564226 10.1186/1471-2334-14-102 PMC3936940

[mbo370332-bib-0010] CLSI . 2025. *Performance Standards for Antimicrobial Susceptibility Testing*. Report No.: CLSI Supplement M100. CLSI.

[mbo370332-bib-0011] Curcio, D. , and F. Fernández . 2007. “Tigecycline Disk Diffusion Breakpoints of *Acinetobacter* spp.: A Clinical Point of View.” Journal of Clinical Microbiology 45, no. 6: 2095–2096. 17548459 10.1128/JCM.00107-07 PMC1933103

[mbo370332-bib-0012] da Silva, K. E. , W. G. Maciel , J. Croda , et al. 2018. “A High Mortality Rate Associated With Multidrug‐Resistant *Acinetobacter baumannii* ST79 and ST25 Carrying OXA‐23 in a Brazilian Intensive Care Unit.” PLoS One 13, no. 12: e0209367. 30592758 10.1371/journal.pone.0209367 PMC6310363

[mbo370332-bib-0013] Deng, M. , M.‐H. Zhu , J.‐J. Li , et al. 2014. “Molecular Epidemiology and Mechanisms of Tigecycline Resistance in Clinical Isolates of *Acinetobacter baumannii* From a Chinese University Hospital.” Antimicrobial Agents and Chemotherapy 58, no. 1: 297–303. 24165187 10.1128/AAC.01727-13 PMC3910737

[mbo370332-bib-0014] Gaddy, J. A. , A. P. Tomaras , and L. A. Actis . 2009. “The *Acinetobacter baumannii* 19606 OmpA Protein Plays a Role in Biofilm Formation on Abiotic Surfaces and in the Interaction of This Pathogen With Eukaryotic Cells.” Infection and Immunity 77, no. 8: 3150–3160. 19470746 10.1128/IAI.00096-09 PMC2715673

[mbo370332-bib-0015] Gedefie, A. , W. Demsiss , M. A. Belete , et al. 2021. “ *Acinetobacter baumannii* Biofilm Formation and Its Role in Disease Pathogenesis: A Review.” Infection and Drug Resistance 14: 3711–3719. 34531666 10.2147/IDR.S332051 PMC8439624

[mbo370332-bib-0016] Ghamari, M. , F. Jabalameli , S. Afhami , S. Halimi , M. Emaneini , and R. Beigverdi . 2024. “ *Acinetobacter baumannii* Infection in Critically Ill Patients With COVID‐19 From Tehran, Iran: The Prevalence, Antimicrobial Resistance Patterns and Molecular Characteristics of Isolates.” Frontiers in Cellular and Infection Microbiology 14: 1511122. 39958989 10.3389/fcimb.2024.1511122 PMC11827423

[mbo370332-bib-0017] Ghasemi, E. , Z. Ghalavand , H. Goudarzi , et al. 2018. “Phenotypic and Genotypic Investigation of Biofilm Formation in Clinical and Environmental Isolates of *Acinetobacter baumannii* .” Archives of Clinical Infectious Diseases 13, no. 4: e12914.

[mbo370332-bib-0018] Ghimire, U. , R. Kandel , M. Neupane , et al. 2021. “Biofilm Formation and blaOXA Genes Detection Among *Acinetobacter baumannii* From Clinical Isolates in a Tertiary Care Kirtipur Hospital, Nepal.” Progress In Microbes & Molecular Biology 4, no. 1. 10.36877/pmmb.a0000245.

[mbo370332-bib-0019] Giske, C. G. , J. Turnidge , R. Cantón , and G. Kahlmeter . 2022. “Update From the European Committee on Antimicrobial Susceptibility Testing (EUCAST).” Journal of Clinical Microbiology 60, no. 3: e0027621. 34346716 10.1128/jcm.00276-21 PMC8925892

[mbo370332-bib-0020] Hameed, F. , M. A. Khan , H. Muhammad , T. Sarwar , H. Bilal , and T. U. Rehman . 2019. “Plasmid‐Mediated mcr‐1 Gene in *Acinetobacter baumannii* and *Pseudomonas aeruginosa*: First Report From Pakistan.” Revista da Sociedade Brasileira de Medicina Tropical 52: e20190237. 31508785 10.1590/0037-8682-0237-2019

[mbo370332-bib-0021] Hamidian, M. , K. E. Holt , D. Pickard , and R. M. Hall . 2016. “A Small *Acinetobacter* Plasmid Carrying the tet39 Tetracycline Resistance Determinant.” Journal of Antimicrobial Chemotherapy 71, no. 1: 269–271. 26416779 10.1093/jac/dkv293 PMC4681370

[mbo370332-bib-0022] Ibrahim, S. , N. Al‐Saryi , I. M. S. Al‐Kadmy , and S. N. Aziz . 2021. “Multidrug‐Resistant *Acinetobacter baumannii* as an Emerging Concern in Hospitals.” Molecular Biology Reports 48, no. 10: 6987–6998. 34460060 10.1007/s11033-021-06690-6 PMC8403534

[mbo370332-bib-0023] Jolley, K. A. , J. E. Bray , and M. C. J. Maiden . 2018. “Open‐Access Bacterial Population Genomics: BIGSdb Software, the PubMLST.org Website and Their Applications.” Wellcome Open Research 3: 124. 30345391 10.12688/wellcomeopenres.14826.1 PMC6192448

[mbo370332-bib-0024] Khoshnood, S. , N. Sadeghifard , N. Mahdian , et al. 2023. “Antimicrobial Resistance and Biofilm Formation Capacity Among *Acinetobacter baumannii* Strains Isolated From Patients With Burns and Ventilator‐Associated Pneumonia.” Journal of Clinical Laboratory Analysis 37, no. 1: e24814. 36573013 10.1002/jcla.24814 PMC9833984

[mbo370332-bib-0025] Khoshnood, S. , M. Savari , E. Abbasi Montazeri , and A. Farajzadeh Sheikh . 2020. “Survey on Genetic Diversity, Biofilm Formation, and Detection of Colistin Resistance Genes in Clinical Isolates of *Acinetobacter baumannii* .” Infection and Drug Resistance 13, no. null: 1547–1558. 32547124 10.2147/IDR.S253440 PMC7266307

[mbo370332-bib-0026] Ko, S. Y. , N. Kim , S. Y. Park , S. Y. Kim , M. Shin , and J. C. Lee . 2023. “ *Acinetobacter baumannii* Under Acidic Conditions Induces Colistin Resistance Through PmrAB Activation and Lipid A Modification.” Antibiotics 12, no. 5: 813. 37237716 10.3390/antibiotics12050813 PMC10215926

[mbo370332-bib-0027] Kobayashi, T. , T. Morimoto , M. Sonohata , and M. Mawatari . 2021. “Is Dislocation Following Total Hip Arthroplasty Caused While Suffering From Delirium?.” Nagoya Journal of Medical Science 83, no. 3: 601–607. 34552292 10.18999/nagjms.83.3.601 PMC8438008

[mbo370332-bib-0028] Kwon, H. I. , S. Kim , M. H. Oh , et al. 2017. “Outer Membrane Protein A Contributes to Antimicrobial Resistance of *Acinetobacter baumannii* Through the OmpA‐Like Domain.” Journal of Antimicrobial Chemotherapy 72, no. 11: 3012–3015. 28981866 10.1093/jac/dkx257

[mbo370332-bib-0029] Li, S. , X. Duan , Y. Peng , and Y. Rui . 2019. “Molecular Characteristics of Carbapenem‐Resistant *Acinetobacter* spp. From Clinical Infection Samples and Fecal Survey Samples in Southern China.” BMC Infectious Diseases 19, no. 1: 900. 31660862 10.1186/s12879-019-4423-3 PMC6819553

[mbo370332-bib-0030] Lin, M.‐F. , Y.‐Y. Lin , H.‐W. Yeh , and C.‐Y. Lan . 2014. “Role of the BaeSR Two‐Component System in the Regulation of *Acinetobacter baumannii* adeAB Genes and Its Correlation With Tigecycline Susceptibility.” BMC Microbiology 14: 119. 24885279 10.1186/1471-2180-14-119 PMC4101873

[mbo370332-bib-0031] Lucaßen, K. , K. Xanthopoulou , J. Wille , et al. 2021. “Characterization of Amino Acid Substitutions in the Two‐Component Regulatory System AdeRS Identified in Multidrug‐Resistant *Acinetobacter baumannii* .” mSphere 6, no. 6: e00709–e00721. 34817237 10.1128/msphere.00709-21 PMC8612257

[mbo370332-bib-0032] Mea, H. J. , P. V. C. Yong , and E. H. Wong . 2021. “An Overview of *Acinetobacter baumannii* Pathogenesis: Motility, Adherence and Biofilm Formation.” Microbiological Research 247: 126722. 33618061 10.1016/j.micres.2021.126722

[mbo370332-bib-0033] Mendes, S. G. , S. I. Combo , T. Allain , S. Domingues , A. G. Buret , and G. J. Da Silva . 2023. “Co‐Regulation of Biofilm Formation and Antimicrobial Resistance in *Acinetobacter baumannii*: From Mechanisms to Therapeutic Strategies.” European Jurnal of Clinical Microbiology & Infectious Diseases 42, no. 12: 1405–1423. 10.1007/s10096-023-04677-8 PMC10651561 37897520

[mbo370332-bib-0034] Mirzaei, B. , Z. N. Bazgir , H. R. Goli , F. Iranpour , F. Mohammadi , and R. Babaei . 2020. “Prevalence of Multi‐Drug Resistant (MDR) and Extensively Drug‐Resistant (XDR) Phenotypes of *Pseudomonas aeruginosa* and *Acinetobacter baumannii* Isolated in Clinical Samples From Northeast of Iran.” BMC Research Notes 13, no. 1: 380. 32778154 10.1186/s13104-020-05224-w PMC7418330

[mbo370332-bib-0035] Monfared, A. M. , A. Rezaei , F. Poursina , and J. Faghri . 2019. “Detection of Genes Involved in Biofilm Formation in MDR and XDR *Acinetobacter baumannii* Isolated From Human Clinical Specimens in Isfahan, Iran.” Archives of Clinical Infectious Diseases 14, no. 2: e85766. 10.5812/archcid.85766.

[mbo370332-bib-0036] Montaña, S. , E. Vilacoba , G. M. Traglia , et al. 2015. “Genetic Variability of AdeRS Two‐Component System Associated With Tigecycline Resistance in XDR‐*Acinetobacter baumannii* Isolates.” Current Microbiology 71: 76–82. 25941024 10.1007/s00284-015-0829-3

[mbo370332-bib-0037] Mustapha, M. M. , B. Li , M. P. Pacey , et al. 2018. “Phylogenomics of Colistin‐Susceptible and Resistant XDR *Acinetobacter baumannii* .” Journal of Antimicrobial Chemotherapy 73, no. 11: 2952–2959. 30124845 10.1093/jac/dky290 PMC6198730

[mbo370332-bib-0038] Ni, W. , X. Cai , B. Liang , Y. Cai , J. Cui , and R. Wang . 2014. “Effect of Proton Pump Inhibitors on In Vitro Activity of Tigecycline Against Several Common Clinical Pathogens.” PLoS One 9, no. 1: e86715. 24466210 10.1371/journal.pone.0086715 PMC3897737

[mbo370332-bib-0039] Novović, K. , and B. Jovčić . 2023. “Colistin Resistance in *Acinetobacter baumannii*: Molecular Mechanisms and Epidemiology.” Antibiotics 12, no. 3: 516. 36978383 10.3390/antibiotics12030516 PMC10044110

[mbo370332-bib-0040] Ouyang, Z. , W. He , M. Jiao , et al. 2024. “Mechanistic and Biophysical Characterization of Polymyxin Resistance Response Regulator Pmra in *Acinetobacter baumannii* .” Frontiers in Microbiology 15: 1293990. 10.3389/fmicb.2024.1293990. 38476937 PMC10927774

[mbo370332-bib-0041] Özkul, C. , and G. Hazırolan . 2021. “Oxacillinase Gene Distribution, Antibiotic Resistance, and Their Correlation With Biofilm Formation in *Acinetobacter baumannii* Bloodstream Isolates.” Microbial Drug Resistance 27, no. 5: 637–646. 32991256 10.1089/mdr.2020.0130

[mbo370332-bib-0042] Palmieri, M. , M. D'Andrea , A. C. Pelegrin , et al., eds. 2019. “Genomic Epidemiology of Carbapenem‐ and Colistin‐Resistant *Acinetobacter baumannii* From Greece.” In Abstract Book Congresso, 29th European Congress of Clinical Microbiology and Infectious Diseases (ECCMID 2019). European Society of Clinical Microbiology and Infectious Diseases (ESCMID).

[mbo370332-bib-0043] Park, Y. K. , J. Y. Choi , D. Shin , and K. S. Ko . 2011. “Correlation Between Overexpression and Amino Acid Substitution of the PmrAB Locus and Colistin Resistance in *Acinetobacter baumannii* .” International Journal of Antimicrobial Agents 37, no. 6: 525–530. 21497062 10.1016/j.ijantimicag.2011.02.008

[mbo370332-bib-0044] Rahman, M. , and S. Ahmad , eds. 2019. “549. First Report for Emergence of Chromosomal Borne Colistin Resistance Gene mcr‐1 in a Clinical *Acinetobacter baumannii* Isolates From India.” Supplement, Open Forum Infectious Diseases 6, no. S2: S261–S262. 10.1093/ofid/ofz360.618.

[mbo370332-bib-0045] Roy, S. , G. Chowdhury , A. K. Mukhopadhyay , S. Dutta , and S. Basu . 2022. “Convergence of Biofilm Formation and Antibiotic Resistance in *Acinetobacter baumannii* Infection.” Frontiers in Medicine 9: 793615. 35402433 10.3389/fmed.2022.793615 PMC8987773

[mbo370332-bib-0046] Salehi, M. , M. Arabi , H. Khalili , et al. 2025. “Impact of an Antimicrobial Time‐Out Program on Antimicrobial Consumption Rate in Hospitalized Patients: A Quasi‐Experimental Study on the National Antimicrobial Stewardship Program in Iran.” Journal of Pharmaceutical Health Care and Sciences 11, no. 1: 41. 40390037 10.1186/s40780-025-00451-4 PMC12090655

[mbo370332-bib-0047] Sepahvand, S. , M. A. Davarpanah , A. Roudgari , A. Bahador , V. Karbasizade , and Z. Kargar Jahromi . 2017. “Molecular Evaluation of Colistin‐Resistant Gene Expression Changes in *Acinetobacter baumannii* With Real‐Time Polymerase Chain Reaction.” Infection and Drug Resistance 10: 455–462. 29225477 10.2147/IDR.S141196 PMC5708186

[mbo370332-bib-0048] Srisakul, S. , D. L. Wannigama , P. G. Higgins , et al. 2022. “Overcoming Addition of Phosphoethanolamine to Lipid A Mediated Colistin Resistance in *Acinetobacter baumannii* Clinical Isolates With Colistin–Sulbactam Combination Therapy.” Scientific Reports 12, no. 1: 11390. 35794134 10.1038/s41598-022-15386-1 PMC9259700

[mbo370332-bib-0049] Sung, J. Y. , S. H. Koo , S. Kim , and G. C. Kwon . 2016. “Persistence of Multidrug‐Resistant *Acinetobacter baumannii* Isolates Harboring bla OXA‐23 and bap for 5 Years.” Journal of Microbiology and Biotechnology 26, no. 8: 1481–1489. 27221112 10.4014/jmb.1604.04049

[mbo370332-bib-0050] Tafreshi, N. , L. Babaeekhou , and M. Ghane . 2019. “Antibiotic Resistance Pattern of *Acinetobacter baumannii* From Burns Patients: Increase in Prevalence of blaOXA‐24‐Like and blaOXA‐58‐Like Genes.” Iranian Journal of Microbiology 11, no. 6: 502–509. 32148682 PMC7048957

[mbo370332-bib-0051] Thirapanmethee, K. , T. Srisiri‐A‐Nun , J. Houngsaitong , P. Montakantikul , P. Khuntayaporn , and M. Chomnawang . 2020. “Prevalence of OXA‐Type β‐Lactamase Genes Among Carbapenem‐Resistant *Acinetobacter baumannii* Clinical Isolates in Thailand.” Antibiotics 9, no. 12: 864. 33287443 10.3390/antibiotics9120864 PMC7761801

[mbo370332-bib-0052] Tomaras, A. P. , C. W. Dorsey , R. E. Edelmann , and L. A. Actis . 2003. “Attachment to and Biofilm Formation on Abiotic Surfaces by *Acinetobacter baumannii*: Involvement of a Novel Chaperone‐Usher Pili Assembly System.” Microbiology 149, no. 12: 3473–3484. 14663080 10.1099/mic.0.26541-0

[mbo370332-bib-0053] Vijayakumar, S. , S. Rajenderan , S. Laishram , S. Anandan , V. Balaji , and I. Biswas . 2016. “Biofilm Formation and Motility Depend on the Nature of the *Acinetobacter baumannii* Clinical Isolates.” Frontiers in Public Health 4: 105. 27252939 10.3389/fpubh.2016.00105 PMC4877508

[mbo370332-bib-0054] Wang, T. H. , Y. S. Leu , N. Y. Wang , C. P. Liu , and T. R. Yan . 2018. “Prevalence of Different Carbapenemase Genes Among Carbapenem‐Resistant *Acinetobacter baumannii* Blood Isolates in Taiwan.” Antimicrobial Resistance & Infection Control 7: 123. 30338061 10.1186/s13756-018-0410-5 PMC6182870

[mbo370332-bib-0055] Yang, C.‐H. , P.‐W. Su , S.‐H. Moi , and L.‐Y. Chuang . 2019. “Biofilm Formation in *Acinetobacter baumannii*: Genotype‐Phenotype Correlation.” Molecules 24, no. 10: 1849. 31091746 10.3390/molecules24101849 PMC6572253

[mbo370332-bib-0056] Yazdansetad, S. , E. Najari , E. A. Ghaemi , N. Javid , A. Hashemi , and A. Ardebili . 2019. “Carbapenem‐Resistant *Acinetobacter baumannii* Isolates Carrying Blaoxa Genes With Upstream ISAba1: First Report of a Novel OXA Subclass From Iran.” Journal of Global Antimicrobial Resistance 18: 95–99. 30763760 10.1016/j.jgar.2018.12.011

[mbo370332-bib-0057] Yousefi Nojookambari, N. , M. Sadredinamin , R. Dehbanipour , et al. 2021. “Prevalence of β‐Lactamase‐Encoding Genes and Molecular Typing of *Acinetobacter baumannii* Isolates Carrying Carbapenemase OXA‐24 in Children.” Annals of Clinical Microbiology and Antimicrobials 20, no. 1: 75. 34702307 10.1186/s12941-021-00480-5 PMC8549256

[mbo370332-bib-0058] Zeighami, H. , F. Valadkhani , R. Shapouri , E. Samadi , and F. Haghi . 2019. “Virulence Characteristics of Multidrug Resistant Biofilm Forming *Acinetobacter baumannii* Isolated From Intensive Care Unit Patients.” BMC Infectious Diseases 19: 629. 31315572 10.1186/s12879-019-4272-0 PMC6637494

