# Supplementary material for: Antibiotic Resistance and Biofilm Gene Distribution in Colistin‐Resistant Acinetobacter baumannii
Source: Microbiologyopen. 2026 Jun 18;15(3):e70332. doi: 10.1002/mbo3.70332 (PMC13277746; doi:10.1002/mbo3.70332)
Supplement: Supplementary file 1 — Supporting File 1 [file MBO3-15-e70332-s001.docx]

**Antibiotic resistance and biofilm gene distribution in colistin-resistant *Acinetobacter baumannii***

Zainab Amer Hatem^1^, Fadhela Nafaa Kafe^2^, Farkad Hawas Musa^3^, Sarah F. Al-Taie^4^, Nabaa Hisham Ateya^5^, Leqaa Majeed Aziz^6^, Erta Rajabi^7^, Raad N Hasan^8*^

^1^Department of biotechnology College of science, University of Diyala, Iraq

^2^Medical Laboratory Techniques department, College of Health and medical technology, University of Al-maarif, Anbar, Iraq

^3^Department of Biology, College of Education for Pure Sciences, University of Anbar, Ramadi, Iraq

^4^University of Baghdad, College of Science, Department of Biotechnology, Baghdad, Iraq

^5^Biotechnology department, College of Applied Science, Fallujah University, Iraq

^6^College of medicine/ University of Fallujah/ Iraq

^7^Faculty of Medicine, Tehran University of Medical Sciences, Tehran, Iran

^8^Biotechnology and Environmental Center/University of Fallujah, Fallujah, Iraq

***Correspondence:**

Prof. Dr. Raad N Hasan

Biotechnology and Environmental Center/University of Fallujah, Fallujah, Iraq

Email: [raadalhasani@uofallujah.edu.iq](mailto:raadalhasani@uofallujah.edu.iq)

**d**

**Contents**

[**Table S1.** 2](#_Toc222750340)

[**Table S2.** 4](#_Toc222750341)

[**Table S3.** 5](#_Toc222750342)

**Table S1.** List of primers used in this study

| **Gene** | **Primer sequence (5′→3′)** | **Product size (bp ^a^)** | **Reference** |
| --- | --- | --- | --- |
| ***bap*** | F-ATGCCTGAGATACAAATTAT  R-GTCAATCGTAAAGGTAACG | 1449 | This study |
| ***ompA*** | F-GTTAAAGGCGACGTAGACG  R-CCAGTGTTATCTGTGTGACC | 578 | This study |
| ***bla_PER-1_*** | F- ATGAATGTCATTATAAAAGC  R- AATTTGGGCTTAGGGCAGAA | 925 | This study |
| ***csuE*** | F-CATCTTCTATTTCGGTCCC  R- CGGTCTGAGCATTGGTAA | 168 | This study |
| ***pmrA*** | F-ACTGGACATGTTGCACTCTTGT  R-ATGCACTTTTATGAAGTCCCGA | 757 | (1) |
| ***pmrB*** | F-TCGGGACTTCATAAAAGTGCAT  R-CAGTCACAGGTGTTCGTAATT | 722 | (1) |
| ***mcr-1*** | F- CGGTCAGTCCGTTTGTTC  R- CTTGGTCGGTCTGTAGGG | 309 | (2) |
| ***mcr-2*** | F- CAAGTGTGTTGGTCGCAGTT  R- TCTAGCCCGACAAGCATACC | 715 | (3) |
| **IS*Aba1*** | F- CACGAATGCAGAAGTTG  R- CGACGAATACTATGACAC | 549 | (4) |
| ***tet(39)*** | F-CTCCTTCTCTATTGTGGCTA  R-CACTAATACCTCTGG ACATCA | 711 | (5) |
| ***bla_OXA‑51_*** | F-TAA TGC TTT GAT CGG CCT TG  R-TGG ATT GCA CTT CAT CTT GG | 353 | (6) |
| ***bla_OXA‑58_*** | F-AAG TAT TGG GGC TTG TGC TG  R-CCC CTC TGC GCT CTA CAT AC | 599 | (6) |
| ***bla_OXA‑23_*** | F-GAT CGG ATT GGA GAA CCA GA  R-ATT TCT GAC CGC ATT TCC AT | 501 | (6) |
| ***bla_OXA‑24_*** | F-CAAGAGCTTGCAAGACGGACT  R-TCCAAGATTTTCTAGCTTATA | 420 | (7) |
| **Real time PCR Primer sequence** | | | |
| ***pmrA*** | F- ATGACAAAAATCTTGATGATTGAAGAT  R- CCATCATAGGCAATCCTAAATCCA | 175 | (8) |
| ***pmrB*** | F- GAACAGCTGAGCACCCTTTAA  R- ACAGGTGGAACCAGCAAATG | 145 | (8) |
| ***rpoB*** | F- GAGTCTAATGGCGGTGGTTC  R- ATTGCTTCATCTGCTGGTTG | 80 | (8) |
| ***adeB*** | F- AACGGACGACCATCT TTG AGTATT  R- CAG TTG TTC CAT TTC ACG CAT T | 84 | (9) |
| ***adeJ*** | F-GGTCATTAATATCTTTGGC  R-GGTACGAATACCGCTGTCA | 54 | (10) |
| ***adeG*** | F-TTCATCTAGCCAAGCAGAAG  R-GTGTAGTGCCACTGGTTACT | 60 | (10) |
| **MLST Primer sequence** | | | |
| ***gltA*** | F-AATTTACAGTGGCACATTAGGTCCC  R-GCAGAGATACCAGCAGAGATACACG | 722 | (11) |
| ***gyrB*** | F-TGTAAAACGACGGCCAGT  R-CAGGAAACAGCTATGACC | 594 | (11) |
| ***gdhb*** | F- GCT ACT TTT ATG CAA CAG AGC C  R- GTT GAG TTG GCG TAT GTT GTG C | 774 | (11) |
| ***recA*** | F- CCTGAATCTTCYGGTAAAAC  R- GTTTCTGGGCTGCCAAACATTAC | 425 | (11) |
| ***cpn60*** | F- GGT GCT CAA CTT GTT CGT GA  R- CAC CGA AAC CAG GAG CTT TA | 640 | (11) |
| ***gpi*** | F- GAA ATT TCC GGA GCT CAC AA  R- TCA GGA GCA ATA CCC CAC TC | 456 | (11) |
| ***rpoD*** | F- ACC CGT GAA GGT GAA ATC AG  R- TTC AGC TGG AGC TTT AGC AAT | 672 | (11) |

^a^ bp = base pair

**Table S2.** STs and genetic characteristics pattern of colistin and tigecycline-resistant *A. baumannii* isolates

| Patient code | Ward | Sample | City | ST ^b^ | Biofilm | Biofilm-related genes | Antibiogram | MIC ^c^ | Resistance-related genes | Expression level | | | |
| --- | --- | --- | --- | --- | --- | --- | --- | --- | --- | --- | --- | --- | --- |
| Colistin resistant | | | | | | | | | | | | | |
|  | | | | | | | | | | ***pmrA*** | | ***pmrA*** | |
| Ab43 | Surgery | Blood | Qom | 188 | S ^l^ | *bap*, *csuE, ompA* | CTZ ^d^, MER ^e^, CIP f, PTZ ^g^, AMK ^h^, GEN ^i^, TET j, IMI ^k^ | 16 | IS*Aba1, bla*_OXA-23/51/24,_ *pmrA/B, mcr-1* | 41 | | 2.84 | |
| Ab60 | Surgery | Abscess | Tehran | 138 |  |  |  | 8 | IS*Aba1, bla*_OXA-23/51,_ *pmrA/B,* | 45.88 | | 4.85 | |
| Ab73 | ICU ^a^ | Sputum | Tehran | 387 |  |  |  | 16 | IS*Aba1, bla*_OXA-23/51,_ *pmrA/B,* | 43.41 | | 3.29 | |
| Tigecycline resistant | | | | | | | | | | | | | |
|  | | | | | | | | | | ***adeB*** | ***adeJ*** | | ***adeG*** |
| Ab41 | ICU | Blood | Tehran | 2288 | S | *bap*, *csuE* | CTZ, MER, CIP, PTZ, AMK, GEN | 16 | *tetX*, IS*Aba1*, *tet*(39), *bla*_OXA-23/58,_ *pmrA/B* | 53.8 | 7.29 | | 8.84 |
| Ab92 | ICU | Sputum | Tehran | 3337 | W ^m^ | *ompA* | CTZ, MER, PTZ, AMK, GEN | 32 | IS*Aba1*, tet(39), *bla*_OXA-23/51,_ *pmrA/B* | 49.23 | 1.01 | | 5.63 |

^a^ ICU = Intensive care unit, ^b^ ST = Sequence type, ^c^ MIC = Minimum inhibitory concentration, ^d^ CTZ = Ceftazidime; ^e^ MER = Meropenem, ^f^ CIP = Ciprofloxacin, ^g^ PTZ = Piperacillin/tazobactam, ^h^ AMK = Amikacin, ^i^ GEN = Gentamicin, ^j^ TET = Tetracycline, ^k^ IMI = Imipenem, ^l^ S = Strong, ^m^ W = Weak,

**Table S3.** Relationship between the biofilm formation ability and antimicrobial susceptibility

| **Antimicrobial Susceptibility** | | | **Biofilm formation strength** | | | |
| --- | --- | --- | --- | --- | --- | --- |
|  |  |  | **Weak** | **Moderate** | **Strong** | **Negative** |
| **Ceftazidime** | R ^a^ | 144 (100%) | 44 | 53 | 34 | 13 |
|  | I ^b^ | 0 (0%) | 0 | 0 | 0 | 0 |
|  | S ^c^ | 0 (0%) | 0 | 0 | 0 | 0 |
| **Tigecycline** | R | 2 (1.4%) | 1 | 0 | 1 | 0 |
|  | I | 0 (0%) | 0 | 0 | 0 | 0 |
|  | S | 142 (98.6%) | 43 | 53 | 33 | 13 |
| **Piperacillin-Tazobactam** | R | 144 (100%) | 44 | 53 | 34 | 13 |
|  | I | 0 (0%) | 0 | 0 | 0 | 0 |
|  | S | 0 (0%) | 0 | 0 | 0 | 0 |
| **Colistin** | R | 3 (2.1%) | 0 | 0 | 3 | 0 |
|  | I | 0 (0%) | 0 | 0 | 0 | 0 |
|  | S | 141 (97.9%) | 44 | 53 | 31 | 13 |
| **Tetracycline** | R | 76 (52.8%) | 21 | 20 | 34 | 1 |
|  | I | 12 (8.3%) | 5 | 6 | 0 | 1 |
|  | S | 56 (38.9%) | 18 | 27 | 0 | 11 |
| **Amikacin** | R | 96 (66.7%) | 29 | 25 | 34 | 8 |
|  | I | 8 (5.6%) | 3 | 3 | 0 | 2 |
|  | S | 40 (27.8%) | 12 | 25 | 0 | 3 |
| **Ciprofloxacin** | R | 65 (45.1%) | 11 | 20 | 34 | 0 |
|  | I | 0 (0%) | 0 | 0 | 0 | 0 |
|  | S | 79 (54.9%) | 33 | 33 | 0 | 13 |
| **Imipenem** | R | 113 (78.5%) | 20 | 47 | 34 | 12 |
|  | I | 0 (0%) | 0 | 0 | 0 | 0 |
|  | S | 31 (21.5%) | 24 | 6 | 0 | 1 |
| **Meropenem** | R | 118 (81.9%) | 25 | 47 | 34 | 12 |
|  | I | 0 (0%) | 0 | 0 | 0 | 0 |
|  | S | 26 (18.1%) | 19 | 6 | 0 | 1 |
| **Gentamicin** | R | 84 (58.3%) | 22 | 28 | 34 | 0 |
|  | I | 6 (4.2%) | 2 | 4 | 0 | 0 |
|  | S | 54 (37.5%) | 20 | 21 | 0 | 13 |

^a^ R = Resistant, ^b^ I = Intermediate, ^c^ S = Susceptible

1. Haeili M, Kafshdouz M, Feizabadi MM. Molecular Mechanisms of Colistin Resistance Among Pandrug-Resistant Isolates of Acinetobacter baumannii with High Case-Fatality Rate in Intensive Care Unit Patients. Microb Drug Resist. 2018;24(9):1271-6.

2. Seleim SM, Mostafa MS, Ouda NH, Shash RYJSr. The role of pmrCAB genes in colistin-resistant Acinetobacter baumannii. 2022;12(1):20951.

3. Rebelo AR, Bortolaia V, Kjeldgaard JS, Pedersen SK, Leekitcharoenphon P, Hansen IM, et al. Multiplex PCR for detection of plasmid-mediated colistin resistance determinants, mcr-1, mcr-2, mcr-3, mcr-4 and mcr-5 for surveillance purposes. 2018;23(6):17-00672.

4. Mohammadi F, Goudarzi H, Hashemi A, Yousefi Nojookambari N, Khoshnood S, Sabzehali F. Detection of ISAba1 in Acinetobacter baumannii Strains Carrying OXA Genes Isolated From Iranian Burns Patients. Arch Pediatr Infect Dis. 2017;5(2):e39307.

5. Taitt CR, Leski TA, Stockelman MG, Craft DW, Zurawski DV, Kirkup BC, et al. Antimicrobial Resistance Determinants in Acinetobacter baumannii Isolates Taken from Military Treatment Facilities. Antimicrobial Agents and Chemotherapy. 2014;58(2):767-81.

6. Turton JF, Woodford N, Glover J, Yarde S, Kaufmann ME, Pitt TL. Identification of Acinetobacter baumannii by detection of the blaOXA-51-like carbapenemase gene intrinsic to this species. J Clin Microbiol. 2006;44(8):2974-6.

7. Royer S, de Campos PA, Araújo BF, Ferreira ML, Gonçalves IR, Batistão DWdF, et al. Molecular characterization and clonal dynamics of nosocomial bla OXA-23 producing XDR Acinetobacter baumannii. PLoS One. 2018;13(6):e0198643.

8. Seleim SM, Mostafa MS, Ouda NH, Shash RY. The role of pmrCAB genes in colistin-resistant Acinetobacter baumannii. Sci Rep. 2022;12(1):20951.

9. Ahmadi F, Khalvati B, Eslami S, Mirzaii M, Roustaei N, Mazloomirad F, et al. The Inhibitory Effect of Thioridazine on adeB Efflux Pump Gene Expression in Multidrug-Resistant Acinetobacter baumannii Isolates Using Real Time PCR. Avicenna J Med Biotechnol. 2022;14(2):132-6.

10. He X, Lu F, Yuan F, Jiang D, Zhao P, Zhu J, et al. Biofilm formation caused by clinical Acinetobacter baumannii isolates is associated with overexpression of the AdeFGH efflux pump. Antimicrobial agents and chemotherapy. 2015;59(8):4817-25.

11. Bartual SG, Seifert H, Hippler C, Luzon MA, Wisplinghoff H, Rodríguez-Valera F. Development of a multilocus sequence typing scheme for characterization of clinical isolates of Acinetobacter baumannii. J Clin Microbiol. 2005;43(9):4382-90.
